# Assessment of Sport and Physical Recreation Participation for Children and Youth with Disabilities: A Systematic Review

**DOI:** 10.3390/ijerph22040557

**Published:** 2025-04-03

**Authors:** Georgina Leigh Clutterbuck, Eugeenia Wen, Sara Petroccitto

**Affiliations:** School of Health and Rehabilitation Sciences, The University of Queensland, Brisbane 4072, Australia

**Keywords:** disability, participation, sport, physical recreation, assessments, outcome measure, psychometric, physical activity, children

## Abstract

Accurate measurement of participation in sport/physical recreation for children with disabilities is important due to decreased physical activity in this population. This review examines the psychometric properties of relevant assessments. Four databases were searched for studies investigating assessments of participation in sport/physical recreation for children and youth with disabilities. The assessment content was analyzed by the proportion of items relevant to sport/physical recreation and the inclusion of participation elements (attendance or involvement). The evidence quality was evaluated using COnsensus-based Standards for the selection of health status Measurement Instruments (COSMIN) checklists and summarized according to Grading of Recommendations Assessment, Development, and Evaluation (GRADE). Nine assessments (46 papers) met criteria. Only the Children’s Assessment of Participation and Enjoyment measured attendance and involvement. Five assessments measured attendance (12–90% items related to sport/physical recreation) and three context-dependent assessments measured involvement. Only the Measure of Experiential Aspects of Participation and Self-reported Experiences of Activity Settings (involvement) were recommended by GRADE. No assessment adequately measured attendance and involvement in sport/physical recreation for children and youth with disabilities. While existing assessments may continue to be used to measure global participation, a comprehensive assessment of sport and physical recreation should be developed with and evaluated for children and youth with disabilities.

## 1. Introduction

Participation in physical activity is important for health, well-being, and quality of life and is associated with improved physical, social, cognitive, and psychological function, especially for children and youth with disabilities [1,2,3,4,5,6,7,8,9]. Participating in physical activity lowers the risk of physical illnesses such as heart disease and cancer [10,11], mental illnesses such as anxiety and depression [12,13], and decreases overall mortality [14,15]. While all types of physical activity offer benefits, those conducted during leisure time (i.e., sport and physical recreation) provide additional advantages beyond expected short-term physical outcomes. Sport and physical recreation encompass physically active pursuits, with sport involving an element of competition and agreed structure, while physical recreation focuses on enjoyment, health, and wellbeing without competition [16]. Compared to activities in the productivity (i.e., work or school) or self-care domains of participation, sport and physical recreation provide opportunities for social engagement [7,17], cognitive stimulation, confidence building, and self-efficacy development [1,7]. They also foster community inclusion and offer an enjoyable, sustainable pathway for lifelong physical activity participation [18,19]. Since children and youth with disabilities have been shown to spend less time in moderate to vigorous leisure-time physical activity and have greater participation in passive recreation (e.g., watching television) than their typically developing peers [20,21], there is significant scope to address overall low rates of physical activity through the introduction of sport and physical recreation compared to other, often non-negotiable activities such as education and self-care. Measures of leisure-time physical activity participation are important to identify individuals who have low levels of participation in this domain and may benefit from intervention, as well as to monitor the effects of those interventions.

Participation is a complex construct that includes both objective and subjective elements [22,23,24]. Imms’ Family of Participation and Participation-related constructs (fPRC) classifies these as *attendance* (objective) and *involvement* (subjective). Attendance metrics include frequency (how often), duration (how long for), and diversity (e.g., in activity, location, or companions) Imms, Granlund [23]. The research from our group [25] identified five key elements that should be measured when collecting data about attending a sport and physical recreation activity: (i) type of activity (e.g., ball sport vs. racing), (ii) competitive level (e.g., social, local, or elite), (iii) team size (e.g., individual, small, or large team), and (iv) location (e.g., field, indoor court, or pool). The subjective experience of *involvement* during attendance at the activity includes elements of personal experience such as enjoyment, engagement, belongingness, and meaning [22,23,24]. Since participation includes both objective and subjective elements, it is best evaluated through patient reported outcome measures. Where this is not possible due to age or cognitive impairment, proxy report may be used with the understanding that the personal experience of involvement cannot be fully captured by another person. Comprehensively measuring both objective attendance and subjective involvement is particularly important when assessing participation in physical activities, where many of the physical and psychological benefits are achieved through both regular and active involvement in the activity.

Researchers and clinicians working with people with disabilities require valid and reliable measures of sport and physical recreation participation to identify individuals with disability who have low participation and may benefit from intervention and to accurately measure the effectiveness of intervention provided to increase participation. The lack of appropriate physical activity participation assessments is widely acknowledged as a limitation in the literature evaluating physical activity interventions for children with disability [26,27,28,29,30,31,32]. In some instances, researchers have questioned if null results on global participation measures that differ from goal attainment measures of participation are due to assessments that are not responsive to change in specific sport and physical recreation interventions, rather than lack of intervention effectiveness [18,32,33,34]. This is particularly important to rectify in landscapes where funding bodies rely on standardized assessments to allocate support to individuals, such as the National Disability Insurance Scheme in Australia. Despite reviews of physical activity participation assessments for older adults [35], individuals experiencing stroke [36], people using wheelchairs [37], and typically developing children and youth [38,39] and evaluations of global participation assessments for children with disabilities [40,41,42,43,44,45,46], there is a distinct gap in the evaluation of the literature regarding assessment of sport and physical recreation participation for children and youth with disabilities.

When a gold-standard assessment is not available, it is common for clinicians and researchers to look to the broader literature to identify potentially useful assessments. In the case of measuring sport and physical recreation participation for children and youth with disability, this includes participation assessments designed to measure participation in contexts other than sport and physical recreation, such as global participation, and assessments designed for different populations, such as those in a different age range. This review will therefore be purposefully broad. The inclusion of assessments designed for adult populations that have been evaluated in populations that include children or youth with disability and assessments that include a low threshold of sport and physical recreation specific items will allow the evaluation of a wide range of possible assessments, with subsequent recommendations for research and clinical practice specific to children and youth with disability.

## 2. Methods

### 2.1. Registration

The protocol for this systematic review was registered in PROSPERO in 2020 (PROSPERO 2020 CRD42020202794). However, due to unforeseen delays related to the COVID-19 pandemic and changes in researcher availability, the project was temporarily put on hold. The review was later resumed with updated searches conducted to reflect the most current evidence. As part of this process, several modifications were made to the original protocol: the publication date range was extended to include studies up to 2025, the focus was refined to include only children and young people (rather than individuals under 65), and adjustments were made to the research team to ensure the review’s successful completion. These amendments were made to ensure the review remained relevant and aligned with the refined research scope. A protocol for this review was not published.

### 2.2. Search Strategy

Four electronic databases (PubMed, EMBASE, CINAHL, and SPORTdiscus) were searched to identify papers that reported on the validity or reliability of assessments of participation for children and youth with disabilities that included sport and physical recreation items. The search terms were designed to capture the population of interest, (‘disability’), study purpose (‘assessment’ or ‘outcome measure’ or ‘test’), and assessment focus (‘participation’ AND (‘sport’ or ‘physical’ or ‘leisure’). Full search terms are available in Supplementary Material one. Primary searches were limited to papers published from 2010 to January 2025 to identify assessments used in current practice and/or research. Secondary searches using the name of each included assessment identified in the original search and (‘validity’ or ‘reliability’) were conducted to identify additional research published at any time. Reference lists of the included studies and relevant systematic reviews (e.g., [47,48]) were hand searched for papers meeting the inclusion criteria. Responsiveness was not evaluated due to the difficulty in identifying response to intervention in the absence of a gold standard assessment of sport and physical recreation participation, and the novel nature of interventions aiming to improve sport and physical recreation participation, for which effectiveness is still emerging.

### 2.3. Inclusion/Exclusion Criteria

Papers were eligible if they met the following criteria:

1. They reported on assessments that
  - evaluated participation [49], including at least one of the following:
    - Attendance diversity (what activity) +/− frequency (how often, or for how long) or
    - Involvement.
  - They were relevant to sport and physical recreation, either because they included
    - ≥20% sport or physical recreation specific items or
    - a subscale specific to sport and/or physical recreation, regardless of the overall make-up of the assessment.
2. They included original psychometric data on validity or reliability
  - for children or youth with disabilities aged 0–24 years or
  - for any population if the paper reported on the original development of an included assessment.
3. They were full-text papers, written in English and published in peer-reviewed journals.

Papers were excluded if they (1) included assessments that were not reproduceable or did not have an accessible test paper (i.e., the researchers could not access a version of the assessment tool with enough detail to determine the measure’s eligibility); (2) reported on earlier editions of an assessment included in this review, where psychometric data for the newer version was already included; or (3) did not adequately identify the activity being participated in (attendance diversity), for example, only reporting the intensity of physical activity (i.e., light, moderate, or vigorous physical activity). This criterion excluded assessments designed to measure physical activity as a health behavior, as these are typically designed to collect data at a population level rather than measure individual participation.

### 2.4. Data Extraction and Analysis

The papers identified by the searches were uploaded to Covidence, where duplicates were removed. The titles and abstracts were examined by two researchers (two of DF, GC, EW, and SP) to determine if they satisfied the inclusion/exclusion criteria. Full text papers were examined if suitability was unclear. Agreement was reached for all papers. Hand searches of relevant systematic reviews were conducted by the first author, with potentially relevant papers uploaded into Covidence and included in the full-text review.

### 2.5. Assessment Content

The content of each assessment was evaluated by a research student (DF or SP) and checked by the author (GC).

All items in included assessments were evaluated to determine if they related to sport or physical recreation according to the Sports Australia definitions [16]. The proportion of items specific to sport and physical recreation were calculated in total and for each relevant subscale.

#### 2.5.1. Sport

“A human activity involving physical exertion and skill as the primary focus of the activity, with elements of competition where rules and patterns of behaviour governing the activity exist formally through organisations and is generally recognised as a sport.”

(p. 7)

#### 2.5.2. Physical Recreation

“Activities engaged in for the purpose of relaxation, health and wellbeing or enjoyment with the primary activity requiring physical exertion, and the primary focus on human activity.”

(p. 7)

Assessments were classified as measuring *attendance*, defined as ‘being there’, and/or *involvement*, defined as the subjective experience of being ‘in the moment’. Assessments measuring attendance were further classified into subcategories of diversity (including elements such as activity type, level, team size and location) and frequency (how often/how much). Assessments measuring involvement were further classified by the specific elements they measured (e.g., enjoyment, mastery, or engagement) [23,24].

### 2.6. Assessment Psychometrics

Full texts of the included papers were reviewed and analysis of the methodological quality of each paper was completed independently by two raters (GC or DF and SP or EW) using the COnsensus-based Standards for the selection of health Measurement INstruments (COSMIN) risk of bias checklist [50]. A third rater was consulted in the case of disagreement. The psychometric properties of the tests (validity and reliability) were rated across multiple criteria using a 4-point scale (very good, adequate, doubtful, and inadequate), according to the COSMIN guidelines. Evidence was reported as ‘positive’ (supportive of the tool), ‘negative’ (against the tool), ‘conflicting’ (both for and against the tool), or ‘unknown’ (inadequate study rating). The quality of validity and reliability for each article was rated as equal to the worst score in that category.

Overall ratings for the strength of psychometric evidence for each assessment was determined by GRADE as recommended by COSMIN guidelines [50]. Strength of evidence was rated as ‘High-level’ (at least one excellent or two adequate studies), ‘moderate-level’ (at least one adequate or two doubtful studies), ‘low-level’ (at least one doubtful or two inadequate studies) or ‘very low-level’ (one inadequate study). Strength and direction of evidence for people with disabilities is presented in a graphic summary as per previous reviews [51,52,53].

### 2.7. Recommendations

The results of the content analysis were combined with the evaluation of psychometric evidence for each assessment to develop clinical and research recommendations relating to assessments of sports and physical recreation participation for children and youth with disabilities.

## 3. Results

**Figure 1:**
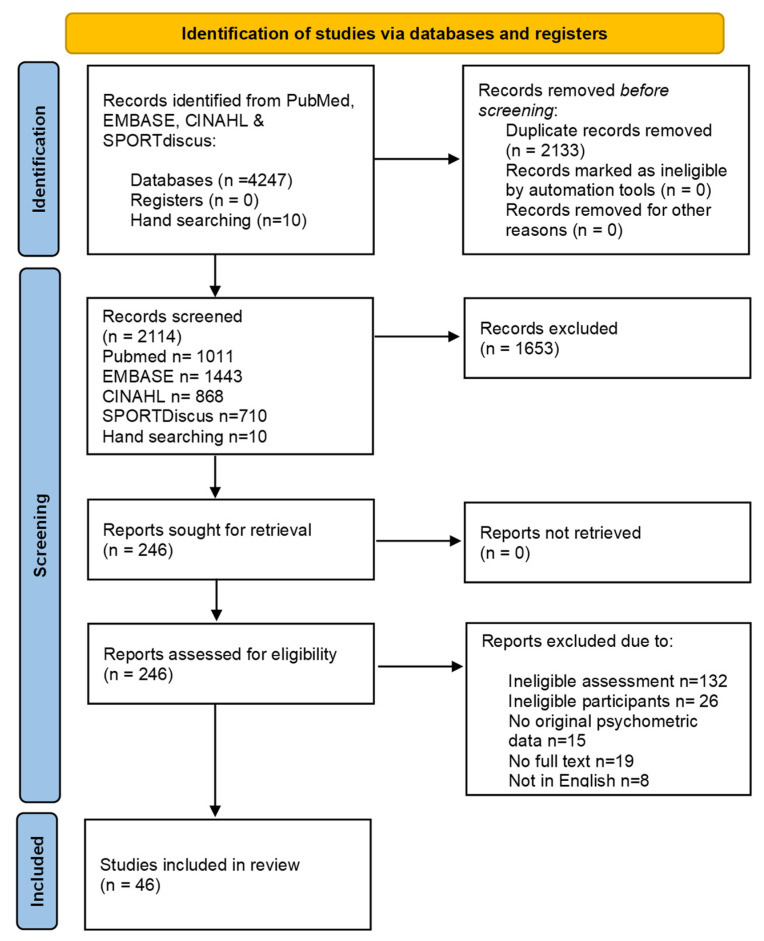
PRISMA Flow Diagram [63].

Database searches identified 1912 original papers published between 2010 and January 2025. Based off a review of titles and abstracts, 282 full-text studies were assessed for eligibility and 36 satisfied the inclusion and exclusion criteria. Secondary searches identified an additional 10 papers for inclusion, with 46 papers evaluated in this review. To confirm that the general terms used (i.e., disability) rather than diagnosis-specific terms (e.g., cerebral palsy, autism, brain injury) captured relevant papers, we compared the yield of a search using disability-specific terms to that of the general term ‘disability’ on PubMed and found no additional papers for inclusion. See the PRISMA flow diagram for details (Figure 1). Nine unique assessments of participation were identified: The Children’s Assessment of Participation and Enjoyment (CAPE) [54], Children’s Leisure Assessment Scale (CLASS) [55], Leisure Assessment Inventory (LAI) [56], Paediatric Activity Card Sort (PACS) [57], Preschool Activity Card Sort (Pre-ACS) [58], Physical Activity Questionnaire (PAQ) for older children or adolescents [59], Measure of Experiential Aspects of Participation (MEAP) [60], Participatory Experience Survey (PES) [61], and Self-reported Experiences of Activity Settings (SEAS) [62].

### 3.1. Excluded Assessments

Fifty-seven assessments were excluded from this review after detailed evaluation of their content at the point of full-text review. Assessment names and reasons for ineligibility are listed in Supplementary Table S1. Most commonly, assessments (i) did not assess participation as defined by the fPRC and instead measured participation-related constructs of activity competence (e.g., Activity Scale for Kids, Rapid assessment of Physical Activity Scale), preferences (e.g., Preference of Activities for Children), or quality of life (e.g., CP Quality of Life); (ii) did not meet the minimum threshold of 20% items relating to sport or physical recreation (e.g., Child and Adolescent Scale of Participation, Participation and Environment Measure for Children and Youth, School Function Assessment); or (iii) did not specify the activity beyond the intensity of physical activity (e.g., International Physical Activity Questionnaire).

### 3.2. Content of Assessments

A summary of the assessments identified with psychometric data for children and youth with disabilities is provided in Table 1.

### 3.3. Populations Evaluated

Due to the broad inclusion criteria, the included studies reported on psychometric properties from a number of different groups of children and youth with disabilities. The assessments were most commonly evaluated for children with cerebral palsy (CAPE, PACS, Pre-ACS, and PAQ) or broader disability groups such as children with physical, intellectual, or developmental disabilities (CLASS, LAI, MEAP, PES, and SEAS). The CAPE was evaluated in the largest range of populations, including children with muscular dystrophy, cerebral palsy, autism, attention deficit hyperactivity disorder, Down syndrome, developmental coordination disorder, spinal cord injury, intellectual disability, and physical disability.

### 3.4. Proportion of Relevant Items

Included assessments ranged from 19–100% of items relevant to sport or physical recreation (CAPE—27%, CLASS—23%, LAI—31%, PACS—24%, Pre-ACS—19%, PAQ—88–90%, MEAP—100%, PES—100%, SEAS—100%). The MEAP, PES, and SEAS are context dependent and therefore report 100% sport and/or physical recreation items when used in this context.

Four assessments included specific subscales relevant to sport and physical recreation, ranging from 50–100% relevant items within each subscale (CAPE—Active physical (69%) and skill-based (50%) subscales, PACS—Sports subscale (100%), Pre-ACS—high physical demand leisure subscale (75%), and CLASS—Games and sports activities subscale (60%)). The CAPE was the only assessment that used subscales to attempt to differentiate between types of physical activities, with both *Active Physical* and *Skill-Based* subscales including primarily sport and physical recreation activities. There were no criteria for the classification of activities into these categories, which limits their utility in the context of assessing sport and physical activity participation. No other assessment included subscales that provided further classification of sport and physical recreational activities (e.g., by categories such as team size, competitive level, location, or type of skills required for the activity).

### 3.5. Participation Construct

In terms of measuring the constructs of attendance and involvement, the CAPE was the only assessment to measure elements of both attendance (frequency and diversity) and involvement (enjoyment only) in sport and physical recreation [54]. The remaining assessments measured attendance frequency and diversity (CLASS, PACS, and PAQ), attendance diversity (LAI and Pre-ACS), or involvement only (MEAP, PES, and SEAS).

### 3.6. Data Type

All assessments collected quantitative data. For attendance, this included ordinal (e.g., frequency) and categorical (e.g., diversity) data. For assessments of involvement that are inherently subjective, this was achieved by imposing binary yes/no or rating scales on participants’ experiences of involvement to achieve a numerical score. Only the SEAS collected qualitative data by asking two open ended questions; however these data were not included in the final score.

### 3.7. Quality of Evidence for Psychometric Properties

Details of the psychometric evidence and individual COSMIN ratings of the included data are provided in Supplementary Tables S2 and S3. Overall strength and direction of evidence, including graphical summaries, are presented in Table S4.

### 3.8. Validity

Evidence of content validity, cross-cultural validity (psychometric data reported only for populations of children with disability), and/or construct validity was available for all assessments (Supplementary Table S2).

Six of the nine assessments reported positive evidence of content validity in the included papers (high-level: CLASS, MEAP, SEAS; moderate-level: CAPE; low-level: Pre-ACS and PES). The PACS, PAQ, and LAI did not have any included studies with evidence of content validity.

Four of the nine assessments reported positive evidence of construct validity for children and youth with disability by discriminating between people with and without disabilities, between subgroups, or comparing scores to outcomes with known or hypothesized relationships to participation (high-level: CLASS, LAI, and MEAP; very low-level: PES). Three assessments reported indeterminate conflicting evidence (high-level: CAPE, Pre-ACS, Moderate-level: PAQ) and one reported negative evidence (high-level: PACS). The CAPE’s conflicting evidence related to high-level positive evidence for overall scores, and high-level negative evidence for sport and physical recreation specific subscales.

While seven assessments were available in other languages (Table 1), only three assessments (CAPE, LAI, and SEAS) had evidence for cross-cultural validity for children and youth with disabilities (Supplementary Table S2). The CAPE demonstrated high-level positive evidence of adaptations to Dutch [69], Spanish [70,80], Portuguese [89,95], Greek [66], Arabic [65], German [72], Swedish [109], and Norwegian [83]. The LAI demonstrated high-level positive evidence for an adaptation to Spanish [104]. The SEAS demonstrated very low-level evidence for a Polish [135] adaptation.

### 3.9. Reliability

Compared to validity, there was more limited evidence available for reliability, with evidence available for only four assessments (CAPE, PAQ, MEAP, and SEAS). Only the CAPE reported inter-rater reliability, and no data were available for intra-rater reliability for children with disability using any assessment in this review (Supplementary Table S3). Three assessments had psychometric evidence for test–retest reliability (high-level: CAPE and SEAS; moderate-level: PAQ; very low-level: PES) and inter-rater reliability (moderate-level: CAPE). Two assessments had positive evidence of internal consistency (high-level: MEAP and SEAS), whereas the CAPE had indeterminate conflicting evidence, with high-level positive evidence for overall scores but high-level negative evidence for sport and physical recreation specific subscales.

### 3.10. GRADE Summary

According to the COSMIN manual for systematic reviews of patient-reported outcome measures, assessments with sufficient content validity (any level of quality of evidence) and sufficient internal consistency with at least low-quality evidence may be recommended for use [50]. The MEAP and SEAS (involvement) were the only assessments in this review to meet these criteria for measuring participation in sports and physical recreation for people with disabilities.

## 4. Discussion

This systematic review identified only nine assessments with a minimum 20% of total items or a subscale specific to participation in sports and physical recreation. None of these assessments were developed as specific measures of participation in sport and physical recreation for children with disabilities. Only one assessment measured both attendance and involvement (CAPE). Five assessments (CLASS, PACS, Pre-ACS, PAQ, and LAI) measured attendance only, with only the PAQ including items that predominately measured participation in sport and physical recreation (88–90%). The three assessments (MEAP, PES, and SEAS) of involvement were context dependent and therefore measured only sport/physical recreation participation when used in this context. No assessment was identified that measured both constructs of participation (attendance and involvement) and included a high proportion of sport- and physical-recreation-specific items.

The availability of psychometrically sound assessments that measure sport and physical recreation participation for people with disabilities is essential clinically to identify individuals requiring intervention to improve participation and to assess the effectiveness of that intervention for individuals, but also in research, to ensure that the evidence reported relating to the effectiveness of interventions aiming to improve participation in sport and physical recreation for children with disabilities is accurate and reliable [136]. None of the assessments identified in this review were developed to measure participation in sport/physical recreation for children with disabilities. Therefore, the psychometric evidence presented needs to be interrogated further to evaluate if it should be used in this context.

Almost all assessments of attendance (CAPE, CLASS, LAI, PACS, and Pre-ACS) were designed as measures of global participation and therefore included low levels of sports- and physical-recreation-specific items (19–31%). The content validity reported for these assessments therefore relates to how well they measure global participation, not sport- and physical-recreation-specific participation. Only the ASQ was developed to evaluate sport and physical recreation participation; however, it had limited data for children with disabilities due to its development for typically developing children in a school context, and there was no evidence of its construct validity. Assessments that included subscales often had sport and physical recreation items across multiple subscales (e.g., CAPE active physical and skill-based subscales). Subscales were rarely evaluated separately; however, when they were, subscales tended to demonstrate negative construct validity and internal consistency [70,85,137], meaning they should not be used in isolation. None of the included assessments collected data relating to activity type, competitive level, team size, or location. The low proportion of items related to sport and/or physical recreation and lack of detail regarding categorization of activities indicates that while global participation measures may be used to measure overall participation that includes a component of sport and physical recreation, they should not be used to measure participation in sport and physical recreation alone.

Measures of involvement in this review were also not explicitly designed to measure participation in sport and physical recreation. However, as the personal experience of involvement is a subjective experience that transcends context, the evidence of content validity reported in this review may be more applicable to measuring participation in sport and physical recreation for assessments of involvement than for measures of attendance. Where the CAPE measures a narrow element of participation (enjoyment), involvement-specific assessments used a variety of conceptual models [22,138] to identify a range of elements to measure. For example, based on a review of conceptualization of participation in physical activity, Martin Ginis, Evans [22] proposed the following characteristics of involvement, on which the MEAP was then developed: autonomy, belongingness, challenge, engagement, mastery, and meaning. Involvement may also be evaluated for a specific occasion of participation (SEAS: “For each question we would like you to tell us how you felt while doing the activity”, PES: “Did you like being at [Activity] today?”) or an activity in general (CAPE: “how much do you like or enjoy doing this activity”, MEAP: “When engaging in [activity], I feel…”).

It is notable that only one measure of involvement collected any qualitative data. While the inherent nature of attendance aligns naturally to quantitative data, measures of involvement imposed artificial rating scales to quantify participants’ subjective experiences. This approach facilitates population comparisons and intervention evaluations; however, it lacks the depth and nuance that qualitative methods can provide. This review’s requirement of a structured, repeatable assessment with an accessible test paper made it unlikely that exclusively qualitative measures would be identified in this review. By their nature, qualitative methods are often tailored to specific contexts and participants. While the measures included in this review offer benefits in terms of simplicity of scoring and statistical analysis, researchers wanting to evaluate involvement in sport and physical recreation should not exclude the types of qualitative methods capable of producing rich, nuanced data, such as interviews, focus groups, or personal reflections using a range of communication methods such as verbal descriptions, text, images, or video (e.g., [139,140]).

In the absence of standardized assessments specific to sport and physical recreation, outcomes that use individualized goals to assess improvements in participation are common [141]. The COPM and GAS have both been shown to be valid and reliable for children with disabilities [40,142,143,144] and have therefore been used to develop and measure change in children’s specific goals, such as those relating to sport and physical recreation [141,145,146]. However, the specific and context-dependent nature of goals poses challenges when used to evaluate long-term effectiveness [147]. Goals are frequently used in clinical practice due to their relevance to individuals’ unique interests, strengths, and challenges. Goal-oriented assessments were not included in this review as changes that are made to assessment protocols to use these assessments in a specific context (such as sport and physical recreation) are not consistent between studies and rarely described in detail.

## 5. Limitations

This review is the first to evaluate assessments of sport and physical recreation for children with disabilities; however, there are some limitations to be considered. First, to ensure that the included assessments could be immediately used by clinicians and researchers working in English-speaking contexts, assessments were excluded if they did not have an English test paper. This may have resulted in assessments used in other languages being excluded from the review, especially if they have been recently developed. Second, we endeavored to use broad search terms to capture a range of literature. However, language around the construct of participation is continually developing. There are a wide range of terms used to describe elements of participation, especially the subjective experience of involvement (e.g., experience, enjoyment, motivation, engagement), and therefore, papers that reported on elements of participation but did not include the participation terms included in our searches and were not identified during hand searching of relevant papers may not have been identified. Similarly, our use of the term ‘disability’, rather than including diagnosis specific terms, may have meant papers that did not directly reference disability were overlooked. Third, psychometric evidence reported in user manuals and other non-peer reviewed publications were not included, meaning that these data (such as content validity of the CAPE) were not reported. Finally, to ensure that all potentially relevant assessments were included in this review, we used broad inclusion criteria, such as a low threshold of physical recreation items. This led to the inclusion of measures that lacked the sensitivity to accurately capture meaningful participation levels among children with disabilities.

### Future Directions

The absence of an assessment that comprehensively assesses sport and physical recreation participation indicates that there is a need for the development of a new, targeted assessment for people with disabilities. Importantly, any new assessment should be developed in collaboration with people with lived experience of disability so that it accurately captures their experiences of participation in sport and physical activity, particularly when measuring the highly personal and subjective experience of involvement.

Based on our review of the development and content of the available assessments and the psychometric properties of the assessments, and our preliminary findings from a Delphi study identifying key constructs for assessing sport and physical recreation participation among children and youth with disabilities [25], the following recommendations are given for the development of a new assessment of participation in sports and physical recreation. The newly developed assessment should be (1) specific to leisure-time physical activity, i.e., sport and physical recreation; (2) measure both attendance (frequency and diversity) and involvement; (3) contain subscales specific to different types of sport and physical recreation (e.g., individual versus team and competitive versus social); (4) be developed at a reading level appropriate for children and/or people with disabilities; (5) be assessed thoroughly in children and youth with disabilities; and (6) be able to measure very low and high levels of participation (i.e., not contain floor or ceiling effects).

This review also identified areas where future research can be directed to increase our understanding of best usage for current assessments. First, a further scoping review of measures of sport and physical recreation participation used in practice but not identified in this research due to their use in other populations or absence of psychometric data may identify tools that could be evaluated. Second, the MEAP, PES, and SEAS have the potential to be accurate and in-depth measures of involvement in sport and physical recreation for children and youth with disabilities; however, they require further evaluation in larger populations of children and youth with a variety of disabilities in this context. Finally, standardized methods are needed so that goal-oriented outcomes may be used consistently to measure sport and physical recreation participation specific contexts, and so that their psychometric properties may be evaluated for children and youth with disability in this context.

## 6. Conclusions

This paper highlights the absence of a comprehensive assessment for sport and physical recreation participation for children and youth with disabilities, emphasizing the need for a targeted tool. Of the nine identified assessments, the MEAP and SEAS were the only assessments recommended by GRADE and may therefore be used to evaluate the participation construct of involvement in this context. Most assessments were not specific to sports and physical recreation, and no assessment comprehensively measured both attendance and involvement. We recommend that a new assessment is codesigned with people with childhood-onset disability to specifically measure sport and physical recreation participation. This tool should measure both attendance (frequency and diversity) and involvement, include subscales for diversity (e.g., activity type, level, environment, team size), and be accessible in language and design for children and people with a range of disabilities.

## Figures and Tables

**Table 1:** Summary of Assessments of Participation in Sports and Physical Recreation for Children and Youth with Disabilities.

Assessment Name	Participation Component	Included Studies	Administration (Mode, Time)	Target Population	Cultural Adaptations	Cost (AUD)	Sport/Physical Recreation Items (%)	Relevant Subscales
**Assessments of attendance and involvement**								
**Children’s Assessment of Participation and Enjoyment (CAPE) [64]**	Attendance (Frequency and Diversity) Involvement (Enjoyment)	[54,65,66,67,68,69,70,71,72,73,74,75,76,77,78,79,80,81,82,83,84,85,86,87,88,89,90,91,92,93,94,95,96,97,98,99]	Self/parent-administered or interview 30–45 min	6–21 years, non-disabled or people with physical or intellectual disability	Arabic [65], Brazilian Portuguese [98], Chinese [100], Dutch [69], German [72], Greek [66], Norwegian [83], Portuguese [89,95], Spanish [80], Swedish [93]	$289 (Test kit and forms)	27% (15/55 items)	Active physical (9/13 items) Skill-based (5/10 items)
**Assessments of attendance**								
**Children’s Leisure Assessment Scale (CLASS) [55]**	Attendance (Frequency and Diversity)	[55,101,102]	Self-administered 15 min	10–18 years, non-disabled or people with physical or intellectual disability	Developed in Hebrew [55]	No Cost (on request from authors)	23% (7/30 items)	Games and Sports (3/5 items)
**Leisure Assessment Inventory (LAI) [56]**	Attendance (Diversity)	[103,104,105]	Interview 45 min	>30 years, non-disabled or people with ID	Spanish [104]	$337 (Test kit and forms)	31% (16/52 items)	No relevant subscale
**Paediatric Activity Card Sort (Pre-ACS) [57]**	Attendance (Frequency and Diversity)	[106]	Parent interview 30–60 min	5–14 years, non-disabled or people with physical or intellectual disability	Japanese [107]	$134 (Test kit and forms)	24% (18/75 items)	Sports (12/12 items)
**Preschool Activity Card Sort (PACS) [58]**	Attendance (Diversity)	[58,108]	Parent interview 30–60 min	3–6 years, non-disabled or people with ID	Arabic (95 activities) [109], Brazilian Portuguese [110], Spanish [111]	$70 (Test kit and forms)	19% (16/85 items)	High physical demand leisure (9/12 items)
**Physical Activity Questionnaire (PAQ) for Older Children or Adolescents [59]**	Attendance (Frequency and Diversity)	[9,112,113]	Self-administered 20 min	8–14 years (PAQ-C) 14–19 years (PAQ-A)	Arabic [114], Brazilian Portuguese [115], Czech [116], Chinese [117,118], Dutch [119], English [120], Ethiopian [121], Gujarati [122], Hungarian [123], Italian [124], Japanese [125], Korean [126], Malay [127], Polish [128], Turkish [129]	No Cost	88–90% (8/9–10 items) The PAQ-A removes one question relating to ‘recess’.	No relevant subscale
**Assessments of involvement**								
**Measure of Experiential Aspects of Participation (MEAP) [60]**	Involvement (Autonomy, Belongingness, Challenge, Engagement, Mastery, Meaning)	[60,130]	Self-administered Time not reported	>19 years, people with physical disability	None identified	No Cost	100% (context dependent)	No relevant subscale
**Participatory Experience Survey (PES) [61]**	Involvement (Environment, Skill Development, Experience)	[131,132]	Interview 3 min	14–22 years, people with intellectual disability	None identified	No Cost	100% (context dependent)	No relevant subscale
**Self-reported Experiences of Activity Settings (SEAS) [133]**	Involvement	[133,134,135]	Self-administered Time not reported	13–23 years, non-disabled or people with physical or intellectual disability	Polish [135]	No Cost	100% (context dependent)	No relevant subscale

## Data Availability

The original contributions presented in this study are included in the article/Supplementary Material. Further inquiries can be directed to the corresponding author.

## Supplementary Materials

The following supporting information can be downloaded at: https://www.mdpi.com/article/10.3390/ijerph22040557/s1, Table S1. Excluded Assessments, Table S2. Validity of Assessments of Participation in Sports & Physical Recreation for People with Disabilities [COSMIN score], Table S3. Reliability of Assessments of Participation in Sports & Physical Recreation for People with Disabilities [COSMIN score], Table S4. Summary of Psychometric Properties of Assessments of Participation in Sports & Physical Recreation for People with Disabilities.
