# Supplementary material for: Assessment of Sport and Physical Recreation Participation for Children and Youth with Disabilities: A Systematic Review
Source: Int J Environ Res Public Health. 2025 Apr 3;22(4):557. doi: 10.3390/ijerph22040557 (PMC12027276; doi:10.3390/ijerph22040557)
Supplement: Supplementary file 1 [file ijerph-22-00557-s001.zip › ijerph-3461692-supplementary.pdf]

Supplementary Material One

Search Terms:

Pubmed

- (((Participation[Title/Abstract]) AND (Disability[Title/Abstract])) AND (Assessment[Title/Abstract] OR "Outcome measure"[Title/Abstract] OR test[Title/Abstract])) AND (Sport[Title/Abstract] OR Physical[Title/Abstract] OR Recreation[Title/Abstract])

Sportdiscus

- AB participation AND AB disability AND AB (Assessment OR "outcome measure" OR test) AND AB (Sport OR physical OR recreation)

Cinahl

- AB Participation AND AB disability AND AB (Assessment OR "outcome measure" OR test) AND (Sport OR physical OR recreation)

Embase

- **participation**:ab,ti AND **disability**:ab,ti AND (**assessment**:ab,ti OR **'outcome measure'**:ab,ti OR **test**:ab,ti) AND (**sport**:ab,ti OR **physical**:ab,ti OR **recreation**:ab,ti)

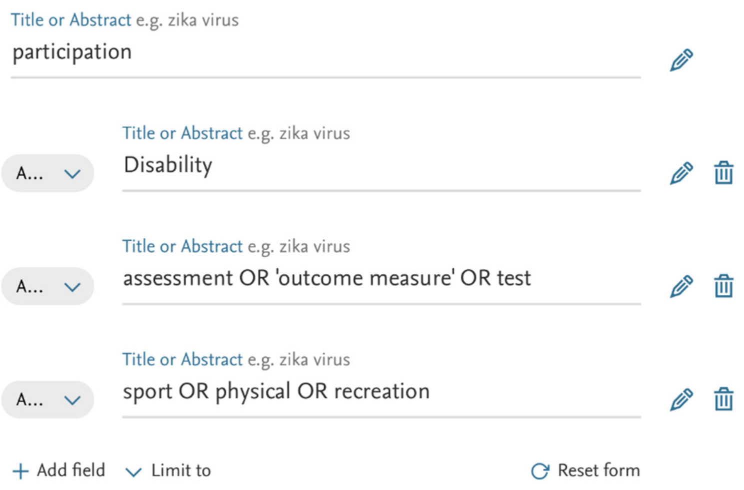

Supplementary Table 1. Excluded Assessments

Reason for exclusion	Assessment
Not participation (activity)	Activity Scale for Kids<br>Assessment of Life Habits<br>School Functional Assessment (Part 2-3)<br>Pediatric Evaluation of Disability Inventory (PEDI)
Not participation (preferences)	Preference of Activities for Children<br>Pediatric Interest Profile<br>ActiveYou
Not participation (quality of life)	CP Quality of Life
Only measures intensity of activity	Physical activity inventory for patients with spinal cord injury<br>7-Day Physical Activity Recall- only intensity of activity<br>Australian Activity Survey- only intensity of activity<br>Brunel lifestyle physical activity questionnaire<br>Global physical activity questionnaire
<20% of items related to sport & physical recreation & no sport/physical recreation specific subscale	Adolescent & Young Adult Activity Card Sort (AYA-ACS)<br>Child & Adolescent scale of Environment<br>Child & Adolescent Scale of Participation (CASP)<br>Children’s Participation Questionnaire<br>Children's Assessment of Participation with H&s

	Children Participation Assessment Scale in Activities<br>Children Helping Out: Responsibilities, Expectations, & Supports<br>Children's Occupational Performance Questionnaire<br>Craig Hospital Inventory of Environmental Factors<br>ICF Measure of Participation and ACTivities (IMPACT-S)<br>Infant Toddler Activity Card Sort<br>Meaningful Activity Participation Assessment<br>Participation & Environment Measure for Children & Youth (PEM-CY) / Youth & Young Adults (Y-PEM)<br>Physical Activity Recall Assessment<br>Picture my Participation<br>Preschool-age Physical Activity Questionnaire<br>Questionnaire of Young People's Participation (QYPP)<br>School Functional Assessment (Part 1)
Did not specify physical activities beyond categories of intensity of PA	Activity monitors (e.g., accelerometers)<br>Godin Leisure-Time Exercise Questionnaire<br>International Physical Activity Questionnaire (long & short form)<br>Leisure Time Physical Activity Questionnaire for People with Spinal Cord Injury<br>Morgenstern Physical Activity Questionnaire<br>Physical Activity Scale for Individuals with Physical Disabilities<br>Physical Activity Recall Assessment for People with Spinal Cord Injury<br>Rapid Assessment of Physical Activity<br>Stanford Brief Activity Survey
Not evaluated in sport/ physical recreation context	Leisure Participation Assessment<br>Pittsburgh Rehabilitation Participation Scale (PRPS)
No psychometric data for children or youth with disability	Activity Card Sort, 2nd edition<br>Activity Questionnaire for Adults and Adolescents (AQuAA)<br>Baecke Questionnaire<br>Minnesota Leisure Time Physical Activity Questionnaire<br>Physical Activity & Disability Scale (PADS18)<br>Physical Activity Scale for the Elderly<br>Short Questionnaire to assess health-enhancing physical activity<br>Yale Physical Activity Survey
Assessment unable to be obtained in English	Activity Participation Assessment<br>Assessment of Preschool Children's Participation<br>Motivation Scale for Sports Participation of People with Disabilities (MSSPPD)<br>The Activities Questionnaire

Supplementary Table S2. Validity of Assessments of Participation in Sports & Physical Recreation for People with Disabilities [COSMIN score]

	Content & Structural validity [evidence direction & level]	Construct validity [evidence direction & level]	Cross-cultural validity [evidence direction & level]
**Assessment of attendance & involvement**			
**CAPE**	[+ Doubtful] Interview with 14 children with autism & 13 TD children. Two new items identified: "going online" & "inventing things" Children asked to explain how they knew their response. 3/14 guessed or didn't know. 3/14 parents disagreed with child's answer [1].<br>[+ Doubtful] Eleven professionals with extensive knowledge & experience with individuals with visual impairments & six dyads of parents & their child with a visual impairment assessed the accuracy of wording for 57 image descriptions & reached consensus in a two-round Delphi study [2].<br>[+Doubtful] Eight researchers & a parent of a child with CP evaluated semantic, idiomatic, experimental & conceptual adequacy of items. Content validity indexes were calculated [3].<br>[+Doubtful] Fifty-one TD children & 8 parents, & 12-18 children with disabilities & 4-6 parents participated in small group interviews to investigate the relevance of the activity items [4].<br>[+Doubtful] Parents of TD children & children with disabilities, three community recreation leaders, two occupational therapists & a psychologist assessed item appropriateness & relevance [5].<br>[+Inadequate] A physiotherapist, paediatrician, neurologist & rehabilitation sciences student & four TD students participated in focus groups regarding the relevance of items [6].	[+Very good] Compared to TD children, children with disabilities had lower total frequency & diversity but not enjoyment (p<0.05) [5].<br>[+Very good] Children with Down syndrome with higher motor & cognitive levels had higher CAPE frequency & diversity (p=0.002-0.004) & enjoyment (p= 0.01) [7].<br>[+Very good] Males with disabilities had higher frequency on the active physical subscale (p=0.001). Females with disabilities have higher frequency on the skill based subscale (p=0.001) [8].<br>[+Very good] CAPE scores (active physical scale frequency) associated with physical-structural environment (CHIEF), classmate support, family income, time constraints, general health (CHQ-50), athletic competence & physical & cognitive & communication functioning in children with disabilities (p<0.01). Frequency of skill-based activities associated with active-recreational & intellectual-cultural orientation (p<0.01). Boys participated in more active physical than girls. With older age, children participated in less recreational but more social activities [9].<br>[+Very good] Children with CP with higher IQ & motor function had higher frequency & intensity of participation on the active physical subscale (p<0.001). Children accessing rehabilitation services had greater frequency & intensity on the skill based subscale (p=0.014-0.015) [10].<br>[+Very good] Compared to TD children, children with physical disabilities had lower frequency & diversity of participation (p=<0.05) [11].<br>[+Very good] Compared to TD children, children with disabilities had lower frequency & diversity on active physical (p<0.001) but not skill-based subscale. Frequency, diversity & enjoyment associated with QoL (p<0.05) [12].<br>[+Very good] Compared to TD children, children with disabilities had lower diversity & intensity of participation in physical activities (p<0.05) [13].<br>[+Very good] Children with disabilities had lower frequency on the active physical & skill-based subscales than TD children (p<0.001) [14].<br>[+Very good] Compared to TD children, children with disabilities had lower total diversity & intensity but not enjoyment (p<0.01) [6].<br>[+Very good] Children with CP had lower frequency & diversity on the skill-based & active physical subscales than TD children (p<0.001) [15].<br>[+Very good] Children with SCI had higher frequency (p<0.001) & enjoyment (p=0.046) on the informal subscales, compared to formal. Frequency on the informal subscale was associated with age, sex & injury level. Intensity on the formal subscale was associated with age & caregiver education [16].<br>[+Very good] Compared to TD children, children with disabilities had lower diversity in active-physical & skill-based subscales (p<0.001) [17].<br>[+Very good] Compared to TD children, children with DCD had lower frequency & diversity but not enjoyment on the active physical subscale (p<0.05) [18].<br>[+ Very Good] Children with more significant neurological injury (SCI) had decreased total frequency & diversity of participation [19].<br>[+ Very Good] Overall participation diversity & intensity (overall) predicted by age (p=.028). Formal participation subscales predicted by GMFCS (p=.001-.015) [20]<br>[+ Very Good] Children with poorer motor function (GMFCS) participated in less activities with friends & others (P=0.01-0.02) [21].<br>[+Very Good] Athletic competence correlated with the active physical subscale (p<.01). Boys participated more frequently than girls in physical activities (p<.01) [22].<br>[+ Very Good] Intensity of participation was associated with gross motor function, socialisation, communication & daily living skills (p<.05) [23]	[+Very good] Translation to/from Spanish. 7 experts assessed cultural relevance. Pilot tested with 7 children with CP & 7 TD children. Compared in a similar group to development (n=199) [12].<br>[+Adequate] Translation to Dutch. 9 experts (OTs, PTs & researchers) assessed cultural relevance & comprehensibility. Compared in a similar group to development (n=74) [14].<br>[+Adequate] Translation to/from Portuguese. Eight experts & one parent assessed cultural relevance. Pilot tested with eight children with CP & eight TD children. Compared in a similar group to development (n=69) [3].<br>[+Adequate] Translation to/ from Arabic. Five experts assessed cultural relevance. Pilot tested in 10 children. Compared in a similar group to development (n=75) [13].<br>[+Doubtful] Forward & back translation to Portuguese, tested on 5 students & discussed with 5 teachers. Five items removed. Then evaluated with 306 children without disabilities & 55 with disabilities [35].<br>[+Doubtful] Translated to/from Swedish. Three new items added, three items excluded [4].<br>[+Doubtful] Translated to Spanish. Two OTs, one psychologist & two other experts reviewed cultural relevance. Pilot tested with parents of disabled & TD children. Compared in similar group to development (n=51) [5].<br>[+Inadequate] Translation to/ from Greek. An OT, PT, psychologist & two recreational readers assessed cultural relevance. Pilot tested with 25 disabled & TD children for comprehensibility. Compared in a similar group to development (n=49) [11].

		[+/-Very good] Compared to TD children, children with autism had lower frequency & diversity on active physical but not skill based subscales, & no difference in enjoyment (p<0.001) [24].<br>[+/-Very good] Compared to TD children, children with ADHD had lower frequency of participation in the skill-based but not active physical subscale (p<0.05) & lower enjoyment in formal activities (p<0.05) [25].<br>[+/-Very good] Compared to TD children, children with CP had lower diversity but not intensity or enjoyment on the active physical & lower intensity but not diversity or enjoyment on skill-based subscale (p<0.05) [26].<br>[+/-Very good] Compared to older children, children with CP 6-12yrs had higher frequency but not diversity or enjoyment on the skill-based (p=0.0001) & diversity & enjoyment but not frequency on the active physical subscale (p=0.01-0.06) [27].<br>[+/-Very good] Compared to TD children, children with ID had lower diversity & frequency (p<0.05) but not enjoyment on skill-based & lower diversity (p<0.05) but not frequency or enjoyment on active physical subscale (p>0.05) [28].<br>[+/-Very good] Compared to TD children, children with DMD had lower frequency on the active physical subscale (p<0.001). There was no difference on the active physical subscale between boys <10 & >10yrs (p>0.05) [29].<br>[+/-Very good] Compared to TD children, children with CP demonstrated lower diversity & frequency (p=0.001) on active physical but not skill-based subscales. Total enjoyment associated psychological well-being [3].<br>[+/- Very Good] Overall & skill-based participation diversity but not active recreation was associated with disability severity (p<.01), Compared to girls, boys scored higher in active physical subscale (p<.01). Children with multiple disabilities were less likely to participate in formal activities (p<.01) [30].<br>[+Doubtful] Compared to girls, boys with disabilities had higher frequency on the active physical subscale & lower frequency on the skill-based subscale (p<0.05). Compared to younger children, children 14–17 years had lower frequency in the physical & skill-based activities (p=0.025) [31].<br>[+Doubtful] Gender related to Physical & Skill-based subscale score (p<0.05) [32].<br>[+/-Doubtful] Compared to TD children, children with disabilities had lower diversity & frequency (p<0.05) but not enjoyment on the skill-based scale & lower frequency (p<0.001) but not diversity or enjoyment on the active physical scale [33].<br>[+/-Doubtful] Compared to TD children, children with DCD had less diversity (p<0.01) & intensity (p<0.05) but not enjoyment in active physical subscale & lower diversity (p<0.01) but not frequency or enjoyment in the skill-based subscale [34].	[+Inadequate] Translation to/from German. A PT, paediatrician, neurologist, & rehab student assessed cultural relevance. Pilot tested with four TD children. Compared in similar group to development (n=32) [6].<br>[+Inadequate] Translation to/from Norwegian. Four experts assessed cultural relevance. Compared in similar group to development (n=34) [36].
**Assessment of attendance**			
**CLASS**	[+Very Good] Five OT's concluded content validity. Five parents reported items were clearly written. Items done by <20% of children eliminated. Factor Analysis identified four distinct factors- Instrumental Indoor Activities, Outdoor Activities with friends or family members, Self-Enrichment Activities & games & Sports Activities. Remaining 10 activities were eliminated [37].	[+Very good] Autistic boys & girls differed in participation variety (p<0.001), frequency (p<0.001) & preferences (p=0.002) [37].<br>[+Very good] Compared to children with TD, children with DCD had lower overall participation diversity (p<0.0001) & frequency (p=0.003) [38].<br>[+Doubtful] Compared to children with TD, children with physical disabilities had lower total frequency & diversity (p<0.05) [39].	No studies met inclusion criteria.
**LAI**	No studies met inclusion criteria.	[+Very good] People with ID & CP participate in more social than leisure activities which are done more than physical activities (p<0.001) [40].<br>[+Very good] Diversity is associated with personal development (p<0.05), self-determination (p<0.01) & social inclusion (p<0.01) (GENCAT) [41].<br>[+Very good] Subjective participation experience correlated with QoL (p>0.05) [42].	[+Very good] Translation to/from Spanish. Four experts assessed cultural relevance. Pilot tested in 15 adults with ID. Compared in similar group to development, n= 231 [41].
**PACS**	No studies met inclusion criteria.	[-Very good] Compared to TD children, children with CP (GMFCSI-II) did not have any difference in diversity on the sport subscale (p>.05) [43].	No studies met inclusion criteria.
**Pre-ACS**	[+ Doubtful] Parents used time logs to identify activities which were categorised by a review of the literature & a subsequent activity list created by two grad students & first author. Ten therapists & ten	[+ Very Good] PAQ scores associated with QoL overall (p<.001) & physical (p<.001), social (p=.022) & psychosocial (p=.022) scales & happiness (p=.015) [45].	No studies met inclusion criteria.

	parents of TD children assessed comprehensibility & relevance of photographs of activities [44].	[-/+Very Good] Compared to TD children, young children with CP demonstrated decreased overall & high-demand leisure participation (p<.001). MACS (p=.01) but not GMFCS was related to participation (p=0.07) [46].	
**PAQ**	No studies met inclusion criteria.	[+Doubtful] Children with physical disability participated less in physical activity than children with intellectual disability (p < .01). Scores were positively associated with self-concept & QoL in both groups [47].<br>[+/- Doubtful] PAQ scores had little to no association with pedometry (r=0.24) or accelerometery (r=0.21), & little to good correlation with self-reported sedentary time (r=-0.03-0.51). PAQ scores associated with GMFCS for adolescents with CP (p<.01), who were less active than their TD peers [48].	No studies met inclusion criteria.
**Assessments of involvement**			
**MEAP**	[+Very good] Three rounds of factor analysis. All items <0.08 for SRMR & RMSEA & >0.90 for CFI & TLI [49].<br>[+Adequate] Literature review for items that measure experience of participation. Items reviewed by 10 sport, employment & mobility experts. Adults with disability tested comprehension [49].	[+Very good] Moderate to large positive associations across elements of the MEAP (r=0.39-0.59) [50].	No studies met inclusion criteria.
**PES**	[+ Doubtful] Six youth with disabilities identified elements of participation which were categorised & coded by research members. Caregivers of youth with developmental disability, allied health & education service providers & cognitive disability experts reviewed content & revised. Accessibility & feasibility evaluated with 8-10 youth with cognitive disabilities [51].	[+ Inadequate] Agreement on positive experiences between the PES & opportunities provided by the setting were high for an art & free swim program (r=0.73) & track & field practice (r=0.80) [52]	No studies met inclusion criteria.
**SEAS**	[+Adequate] Literature review of existing tools used to develop items. Five SP, OT & child psychiatrists reviewed items. Item relevance & compressibility reviewed by experienced teacher & five children with disability [53].<br>[Adequate+] Developed with graphic symbols for AAC users. Eleven SLP & communication assistance professionals & five adult AAC users found SEAS with SEAS-PCS equivalent [54].<br>[+Doubtful] Factor loading <0.50 excluded (12 items); 4/5 scales remained the same. Factor analysis identified five factors accounting for 61.9% of variance: Psychological Engagement, Social Belonging, Meaningful Interactions, Choice & Control & Personal Growth [53].	No studies met inclusion criteria.	[+ Inadequate] Translation to/from Polish. Intelligibility was reported by 89.3% of respondents [55]

TD= Typically developing, OT= Occupational therapist, TBI=traumatic brain injury, CP= Cerebral palsy, PT= Physiotherapist, ADHD= Attention deficit hyperactive disorder, ID= intellectual disability, DMD= Duchenne's muscular dystrophy, DCD= developmental coordination disorder, SP= Speech pathologist, SRMR= Standardized Root Mean Square Residual, RMSEA= Root Mean Square Error of Approximation.

Supplementary Table S3. Reliability of Assessments of Participation in Sports & Physical Recreation for People with Disabilities [COSMIN score]

	Reliability	Internal consistency
**Assessments of attendance & involvement**		
**CAPE**	[+Very good] Test-retest reliability good for all subscales (ICC= 0.74-0.83) [3].<br><br>[+Very good] Test-retest reliability was moderate-good for all subscales for diversity (0.58–0.74) & moderate to excellent for intensity (ICC=0.65-0.82) [6].<br><br>[+Very good] Test-retest reliability was moderate-good for overall diversity (ICC=0.54), intensity (ICC=0.74), with whom (ICC=0.71), where (ICC=0.76) & enjoyment (ICC=0.80) [12].<br><br>[+Very good] Test-retest reliability was moderate-good for overall score (ICC=0.66) & moderate-excellent for all subscales (ICC=0.49-0.83) [36].<br><br>[+Adequate] Test-retest reliability moderate to excellent for overall diversity (ICC=0.733), frequency (ICC=0.752) & enjoyment (ICC=0.758) & all subscales (ICC=0.715-0.752) except social (r=0.196) [1].<br><br>[+ Adequate] Test-retest reliability was moderate to excellent for overall diversity (*r*= 0.67), intensity (*r*= 0.69), with whom (*r*= 0.58), where (*r*= 0.91), & enjoyment (*r* = 0.80) [56].<br><br>[+Adequate] Test-retest (ICC=0.61-0.78) & inter-rater reliability (0.65-0.83) moderate to good in all subscales [14].	[+/-Very good] Good IC for overall score (α= 0.79) & formal (α= 0.75) but poor for active physical (α= 0.48) & skill-based subscales (α=0.40) [3].<br><br>[+/-Very good] Good IC for overall score (α= 0.80) but poor for subscales (α>0.42) [6].<br><br>[+/-Doubtful] Good IC for overall score (α= 0.87) & acceptable (α= 0.71-0.73) for all but the Social & Self-Improvement subscales [36].<br><br>[+Doubtful] Good internal consistency for overall score (α=0.85) [5].<br><br>[+Inadequate] Good IC for overall score (α= 0.83) & physical (α= 0.83) & self-improvement (α= 0.73) subscales [13].<br><br>[-Inadequate] Poor to moderate IC of intensity for subscales (α= 0.08-0.64) [11].
**Assessment of attendance**		
**CLASS**	No studies met inclusion criteria.	No studies met inclusion criteria.
**LAI**	No studies met inclusion criteria.	No studies met inclusion criteria.
**PACS**	No studies met inclusion criteria.	No studies met inclusion criteria.
**Pre-ACS**	No studies met inclusion criteria.	No studies met inclusion criteria.
**PAQ**	[+Adequate] Test-retest reliability was fair to excellent for individual PAQ items (ICC=0.51-0.99), overall PA (ICC=0.90); sedentary items (ICC=0.3-0.95) & total weekly sedentary time (ICC=0.84) [48]	No studies met inclusion criteria.
**Assessments of involvement**		
**MEAP**	No studies met inclusion criteria.	[+Very good] Good IC for autonomy (α=0.78-0.85), belongingness (α=0.82-0.90), challenge (α=0.70-0.82), engagement (α=0.73-0.80), mastery (α=0.77-0.81), meaning (α=0.53-0.64) [49].<br><br>[+Adequate] Excellent IC for overall score (α=0.90) [50].
**PES**	No studies met inclusion criteria.	No studies met inclusion criteria.
**SEAS**	[+Very good] Personal growth (ICC=0.85), Psychological engagement (ICC=0.59), Social belonging (ICC=0.53), Meaningful interactions (ICC=0.94), Choice & control (ICC=0.51) [53].	[+Very Good] Good IC for Personal growth (α=0.86) Psychological growth (α = 0.88), Social belonging (α=0.78), Meaningful interaction (α=0.71), Choice & Control (α=0.78) [53].<br><br>[+Very Good] Excellent overall IC (α=0.953) [55]

PT= Physiotherapist, OT=Occupational therapist, IC=Internal Consistency

Table S4. Summary of Psychometric Properties of Assessments of Participation in Sports & Physical Recreation for People with Disabilities.

	**Validity**						**Reliability**						**Visual Representation**
	Content		Construct		Cross-cultural		Test-retest		Inter/intra-rater		Internal consistency		
	+/-	COSMIN	+/-	COSMIN	+/-	COSMIN	+/-	COSMIN	+/-	COSMIN	+/-	COSMIN	
**Assessments of Attendance & Involvement**													
**CAPE**	+++	6 D	?	19E (+)<br>8E (+/-)<br>2D (+)<br>2D (+/-)	++++	1E<br>3A<br>3D<br>3I	++++	4E<br>3A	+++	1A	?	2E (+/-)<br>1D (+/-)<br>1D (+)<br>1I (+)<br>1I (-)	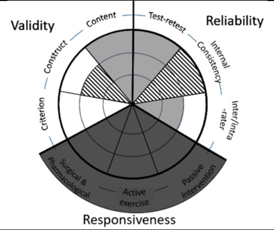
**Assessments of Attendance**													
**PACS**			----	1E									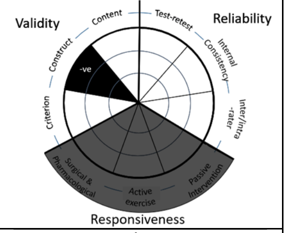
**Pre-ACS**	++	1D	?	1E (+)<br>1E (+/-)									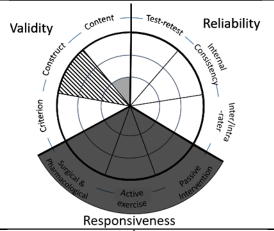
**PAQ**			?	1D (+)<br>1D (+/-)			+++	1A					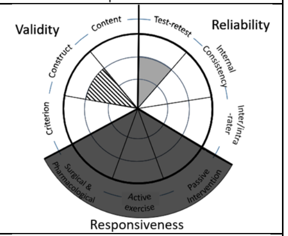
**CLASS**	++++	1E	++++	2E<br>1D									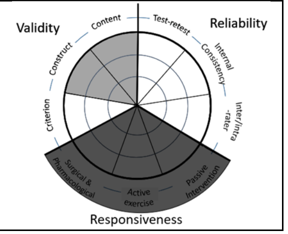

**LAI**			++++	3E	++++	1E							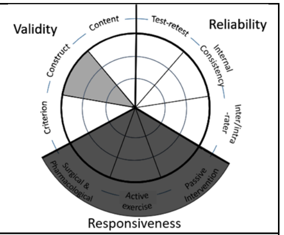
**Assessments of Involvement**													
**MEAP**	++++	1E<br>1A	++++	1E							++++	1E<br>1A	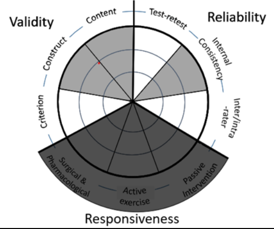
**PES**	++	1D	+	1I									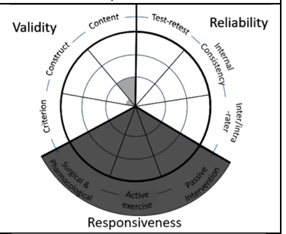
**SEAS**	++++	2A<br>1D			+	1I	++++	1E			++++	2E	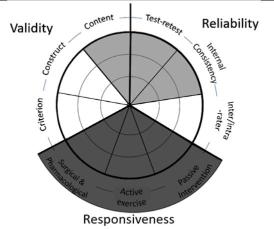

Key

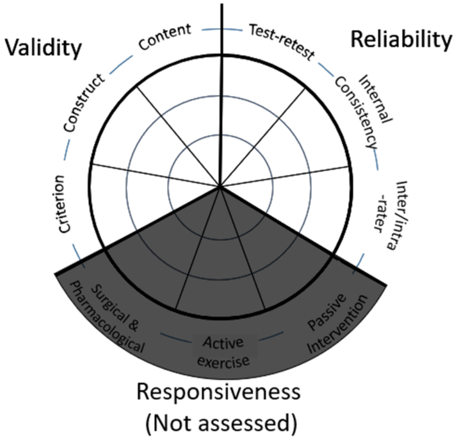

**Strength of Evidence**			
High-level **(++++ or - - - -)**	***Moderate-level*** **(+++ or - - -)**	***Low-level*** **(++ or - -)**	***Very low-level*** **(+ or -)**
>1 excellent, or 2 adequate studies	1 adequate, or 2 doubtful studies	1 doubtful, or 2 inadequate studies	1 inadequate study
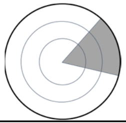	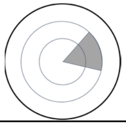	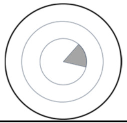	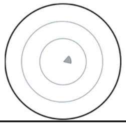
**Direction of Evidence**			
Sufficient/positive (+)	Insufficient/ negative (-)	*indeterminant/ conflicting (?)*	*Not evaluated*
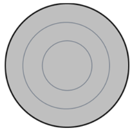	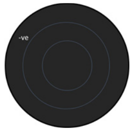	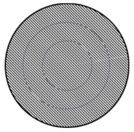	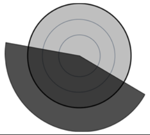

E= Excellent, A= adequate, D= doubtful, I= inadequate

References

1. Potvin, M.C.; Snider, L.; Prelock, P.; Kehayia, E.; Wood-Dauphinee, S. Children's assessment of participation and enjoyment/preference for activities of children: Psychometric properties in a population with high-functioning autism. *Am. J. Occup. Ther.* **2013**, *67*, 209–217. https://doi.org/10.5014/ajot.2013.006288.
2. Perreault, M.; Belknap, K.; Lieberman, L.; Beach, P. Validation of Image Descriptions for the Children's Assessment of Participation and Enjoyment and Preferences for Activities of Children: A Delphi Study. *J. Vis. Impair. Blind.* **2022**, *116*, 736–743. https://doi.org/10.1177/0145482X221129619.
3. Vila-Nova, F.; Oliveira, R.; Cordovil, R. Cross-Cultural Validation of Children's Assessment of Participation and Enjoyment Portuguese Version. *Front. Pediatr.* **2019**, *7*, 33. https://doi.org/10.3389/fped.2019.00033.
4. Ullenhag, A.; Almqvist, L.; Granlund, M.; Krumlinde-Sundholm, L. Cultural validity of the Children's Assessment of Participation and Enjoyment/Preferences for Activities of Children (CAPE/PAC). *Scand. J. Occup. Ther.* **2012**, *19*, 428–438. https://doi.org/10.3109/11038128.2011.631218.
5. Colón, W.I.; Rodríguez, C.; Ito, M.; Reed, C.N. Psychometric evaluation of the Spanish version of the Children's Assessment of Participation and Enjoyment and Preferences for Activities of Children. *Occup. Ther. Int.* **2008**, *15*, 100–113. https://doi.org/10.1002/oti.250.
6. Fink, A.; Gebhard, B.; Erdwiens, S.; Haddenhorst, L.; Nowak, S. Reliability of the German version of the Children's Assessment of Participation and Enjoyment (CAPE) and Preferences for Activities of Children (PAC). *Child Care Health Dev.* **2016**, *42*, 683–691. https://doi.org/10.1111/cch.12360.
7. Wuang, Y.; Su, C.Y. Patterns of participation and enjoyment in adolescents with Down syndrome. *Res. Dev. Disabil.* **2012**, *33*, 841–848. https://doi.org/10.1016/j.ridd.2011.12.008.
8. Law, M.; King, G.; King, S.; Kertoy, M.; Hurley, P.; Rosenbaum, P.; Young, N.; Hanna, S. Patterns of participation in recreational and leisure activities among children with complex physical disabilities. *Dev. Med. Child. Neurol.* **2006**, *48*, 337–342. https://doi.org/10.1017/s0012162206000740.
9. King, G.A.; Law, M.; King, S.; Hurley, P.; Hanna, S.; Kertoy, M.; Rosenbaum, P. Measuring children's participation in recreation and leisure activities: Construct validation of the CAPE and PAC. *Child: Care Health Dev.* **2007**, *33*, 28–39. https://doi.org/10.1111/j.1365-2214.2006.00613.x.
10. Majnemer, A.; Shevell, M.; Law, M.; Birnbaum, R.; Chilingaryan, G.; Rosenbaum, P.; Poulin, C. Participation and enjoyment of leisure activities in school-aged children with cerebral palsy. *Dev. Med. Child. Neurol.* **2008**, *50*, 751–758. https://doi.org/10.1111/j.1469-8749.2008.03068.x.
11. Anastasiadi, I.; Tzetzis, G. Construct validation of the Greek version of the Children's Assessment of Participation and Enjoyment (CAPE) and Preferences for Activities of Children (PAC). *J. Phys. Act. Health* **2013**, *10*, 523–532. https://doi.org/10.1123/jpah.10.4.523.
12. Longo, E.; Badia, M.; Orgaz, B.; Verdugo, M.A. Cross-cultural validation of the Children's Assessment of Participation and Enjoyment (CAPE) in Spain. *Child. Care Health Dev.* **2014**, *40*, 231–241. https://doi.org/10.1111/cch.12012.
13. Almasri, N.A.; Palisano, R.J.; Kang, L.J. Cultural adaptation and construct validation of the Arabic version of children's assessment of participation and enjoyment and preferences for activities of children measures. *Disabil. Rehabil.* **2019**, *41*, 958–965. https://doi.org/10.1080/09638288.2017.1416498.
14. Bult, M.K.; Verschuren, O.; Gorter, J.W.; Jongmans, M.J.; Piskur, B.; Ketelaar, M. Cross-cultural validation and psychometric evaluation of the Dutch language version of the Children's Assessment of Participation and Enjoyment (CAPE) in children with and without physical disabilities. *Clin. Rehabil.* **2010**, *24*, 843–853. https://doi.org/10.1177/0269215510367545.
15. Hassani Mehraban, A.; Hasani, M.; Amini, M. The Comparison of Participation in School-Aged Cerebral Palsy Children and Normal Peers: A Preliminary Study. *Iran. J. Pediatr.* **2016**, *26*, e5303. https://doi.org/10.5812/ijp.5303.
16. Klaas, S.J.; Kelly, E.H.; Gorzkowski, J.; Homko, E.; Vogel, L.C. Assessing patterns of participation and enjoyment in children with spinal cord injury. *Dev. Med. Child. Neurol.* **2010**, *52*, 468–474. https://doi.org/10.1111/j.1469-8749.2009.03552.x.

17. Bult, M.; Verschuren, O.; Lindeman, E.; Jongmans, M.; Ketelaar, M. Do children participate in the activities they prefer? A comparison of children and youth with and without physical disabilities. *Clin. Rehabil.* **2014**, *28*, 388–396. https://doi.org/10.1177/0269215513504314.
18. Fong, S.S.M.; Lee, V.Y.L.; Chan, N.N.C.; Chan, R.S.H.; Chak, W.-K.; Pang, M.Y.C. Motor ability and weight status are determinants of out-of-school activity participation for children with developmental coordination disorder. *Res. Dev. Disabil.* **2011**, *32*, 2614–2623. https://doi.org/10.1016/j.ridd.2011.06.013.
19. Riordan, A.; Kelly, E.H.; Klaas, S.J.; Vogel, L.C. Psychosocial outcomes among youth with spinal cord injury by neurological impairment. *J. Spinal Cord Med.* **2015**, *38*, 76–83. https://doi.org/10.1179/2045772313Y.0000000162.
20. Souto, D.O.; Cardoso de Sa, C.d.S.; de Lima Maciel, F.K.; Vila-Nova, F.; Gonçalves de Souza, M.; Guimarães Ferreira, R.; Longo, E.; Leite, H.R. I Would Like to Do It Very Much! Leisure Participation Patterns and Determinants of Brazilian Children and Adolescents with Physical Disabilities. *Pediatr. Phys. Ther.* **2023**, *35*, 304–312. https://doi.org/10.1097/PEP.0000000000001019.
21. Robert, J.P.; Lin-Ju, K.; Lisa, A.C.; Margo, O.; Donna, O.; Jill, M. Social and Community Participation of Children and Youth With Cerebral Palsy Is Associated With Age and Gross Motor Function Classification. *Phys. Ther.* **2009**, *89*, 1304–1314. https://doi.org/10.2522/ptj.20090162.
22. King, G.A.; Law, M.; King, S.; Hurley, P.; Hanna, S.; Kertoy, M.; Rosenbaum, P. Measuring children's participation in recreation and leisure activities: Construct validation of the CAPE and PAC. *Child: Care Health Dev.* **2007**, *33*, 28–39. https://doi.org/10.1111/j.1365-2214.2006.00613.x.
23. Shikako-Thomas, K.; Shevell, M.; Schmitz, N.; Lach, L.; Law, M.; Poulin, C.; Majnemer, A. Determinants of participation in leisure activities among adolescents with cerebral palsy. *Res. Dev. Disabil.* **2013**, *34*, 2621–2634. https://doi.org/10.1016/j.ridd.2013.05.013.
24. Hilton, C.L.; Crouch, M.C.; Israel, H. Out-of-school participation patterns in children with high-functioning autism spectrum disorders. *Am. J. Occup. Ther.* **2008**, *62*, 554–563. https://doi.org/10.5014/ajot.62.5.554.
25. Shimoni, M.; Engel-Yeger, B.; Tirosh, E. Participation in leisure activities among boys with attention deficit hyperactivity disorder. *Res. Dev. Disabil.* **2010**, *31*, 1234–1239. https://doi.org/10.1016/j.ridd.2010.07.022.
26. Engel-Yeger, B.; Jarus, T.; Anaby, D.; Law, M. Differences in patterns of participation between youths with cerebral palsy and typically developing peers. *Am. J. Occup. Ther.* **2009**, *63*, 96–104. https://doi.org/10.5014/ajot.63.1.96.
27. Majnemer, A.; Shikako-Thomas, K.; Schmitz, N.; Shevell, M.; Lach, L. Stability of leisure participation from school-age to adolescence in individuals with cerebral palsy. *Res. Dev. Disabil.* **2015**, *47*, 73–79. https://doi.org/10.1016/j.ridd.2015.08.009.
28. King, M.; Shields, N.; Imms, C.; Black, M.; Ardern, C. Participation of children with intellectual disability compared with typically developing children. *Res. Dev. Disabil.* **2013**, *34*, 1854–1862. https://doi.org/10.1016/j.ridd.2013.02.029.
29. Bendixen, R.M.; Senesac, C.; Lott, D.J.; Vandenborne, K. Participation and quality of life in children with Duchenne muscular dystrophy using the International Classification of Functioning, Disability, and Health. *Health Qual Life Outcomes* **2012**, *10*, 43. https://doi.org/10.1186/1477-7525-10-43.
30. Shields, N.; Synnot, A.; Kearns, C. The extent, context and experience of participation in out-of-school activities among children with disability. *Res. Dev. Disabil.* **2015**, *47*, 165–174. https://doi.org/10.1016/j.ridd.2015.09.007.
31. Nyquist, A.; Moser, T.; Jahnsen, R. Fitness, Fun and Friends through Participation in Preferred Physical Activities: Achievable for Children with Disabilities? *Int. J. Disabil. Dev. Educ.* **2016**, *63*, 334–356. https://doi.org/10.1080/1034912X.2015.1122176.
32. Ullenhag, A.; Bult, M.K.; Nyquist, A.; Ketelaar, M.; Jahnsen, R.; Krumlinde-Sundholm, L.; Almqvist, L.; Granlund, M. An international comparison of patterns of participation in leisure activities for children with and without disabilities in Sweden, Norway and the Netherlands. *Dev. Neurorehabil* **2012**, *15*, 369–385. https://doi.org/10.3109/17518423.2012.694915.
33. Ullenhag, A.; Krumlinde-Sundholm, L.; Granlund, M.; Almqvist, L. Differences in patterns of participation in leisure activities in Swedish children with and without disabilities. *Disabil. Rehabil.* **2014**, *36*, 464–471. https://doi.org/10.3109/09638288.2013.798360.

34. Jarus, T.; Lourie-Gelberg, Y.; Engel-Yeger, B.; Bart, O. Participation patterns of school-aged children with and without DCD. *Res. Dev. Disabil.* **2011**, *32*, 1323–1331. https://doi.org/10.1016/j.ridd.2011.01.033.
35. Sanches-Ferreira, M.; Alves, S.; Silveira-Maia, M. Translation, Adaptation and Validation of the Portuguese Version of Children's Assessment of Participation and Enjoyment / Preferences for Activities of Children (CAPE / PAC). *J. Occup. Ther. Sch. Early Interv.* **2023**, *16*, 593–607. https://doi.org/10.1080/19411243.2022.2129903.
36. Nordtorp, H.L.; Nyquist, A.; Jahnsen, R.; Moser, T.; Strand, L.I. Reliability of the Norwegian version of the Children's Assessment of Participation and Enjoyment (CAPE) and Preferences for Activities of Children (PAC). *Phys. Occup. Ther. Pediatr.* **2013**, *33*, 199–212. https://doi.org/10.3109/01942638.2012.739269.
37. Rosenblum, S.; Sachs, D.; Schreuer, N. Reliability and validity of the Children's Leisure Assessment Scale. *Am. J. Occup. Ther.* **2010**, *64*, 633–641. https://doi.org/10.5014/ajot.2010.08173.
38. Rosenblum, S.; Waissman, P.; Diamond, G.W. Identifying play characteristics of pre-school children with developmental coordination disorder via parental questionnaires. *Hum. Mov. Sci.* **2017**, *53*, 5–15. https://doi.org/10.1016/j.humov.2016.11.003.
39. Schreuer, N.; Sachs, D.; Rosenblum, S. Participation in leisure activities: Differences between children with and without physical disabilities. *Res. Dev. Disabil.* **2014**, *35*, 223–233. https://doi.org/10.1016/j.ridd.2013.10.001.
40. Badia, M.; Orgaz, M.B.; Verdugo, M.; Ullán, A.M. Patterns and determinants of leisure participation of youth and adults with developmental disabilities. *J. Intellect. Disabil. Res.* **2013**, *57*, 319–332. https://doi.org/10.1111/j.1365-2788.2012.01539.x.
41. Badia, M.; Orgaz-Baz, M.B.; Verdugo, M.A.; Martínez-Aguirre, M.M.; Longo-Araújo-de-Melo, E.; Ullán-de-la-Fuente, A.M. Adaptation and validation of the Spanish version of the Leisure Assessment Inventory. *Intellect. Dev. Disabil.* **2012**, *50*, 233–242. https://doi.org/10.1352/1934-9556-50.3.233.
42. Badia, M.; Orgaz, M.B.; Verdugo, M.; Ullán, A.M.; Martínez, M. Relationships between leisure participation and quality of life of people with developmental disabilities. *J. Appl. Res. Intellect. Disabil.* **2013**, *26*, 533–545. https://doi.org/10.1111/jar.12052.
43. Calley, A.; Williams, S.; Reid, S.; Blair, E.; Valentine, J.; Girdler, S.; Elliott, C. A comparison of activity, participation and quality of life in children with and without spastic diplegia cerebral palsy. *Disabil. Rehabil.* **2012**, *34*, 1306–1310. https://doi.org/10.3109/09638288.2011.641662.
44. Berg, C.; Lavesser, P. The Preschool Activity Card Sort. *OTJR Occup. Particip. Health* **2006**, *26*, 143–151. https://doi.org/10.1177/153944920602600404.
45. Maher, C.A.; Toohey, M.; Ferguson, M. Physical activity predicts quality of life and happiness in children and adolescents with cerebral palsy. *Disabil. Rehabil.* **2016**, *38*, 865–869. https://doi.org/10.3109/09638288.2015.1066450.
46. Abu-Dahab, S.M.N.; Alheresh, R.A.; Malkawi, S.H.; Saleh, M.; Wong, J. Participation patterns and determinants of participation of young children with cerebral palsy. *Aust. Occup. Ther. J.* **2021**, *68*, 195–204. https://doi.org/10.1111/1440-1630.12714.
47. Yang, W.; Yu, J.J.; Wong, S.H.S.; Sum, R.K.W.; Li, M.H.; Sit, C.H.P. The Associations Among Physical Activity, Quality of Life, and Self-Concept in Children and Adolescents with Disabilities: A Moderated Mediation Model. *Front. Pediatr.* **2022**, *10*, 947336. https://doi.org/10.3389/fped.2022.947336.
48. Maher, C.A.; Williams, M.T.; Olds, T.; Lane, A.E. Physical and sedentary activity in adolescents with cerebral palsy. *Dev. Med. Child Neurol.* **2007**, *49*, 450–457. https://doi.org/10.1111/j.1469-8749.2007.00450.x.
49. Caron, J.G.; Martin Ginis, K.A.; Rocchi, M.; Sweet, S.N. Development of the Measure of Experiential Aspects of Participation for People with Physical Disabilities. *Arch. Phys. Med. Rehabil.* **2019**, *100*, 67–77.e62. https://doi.org/10.1016/j.apmr.2018.08.183.
50. Arbour-Nicitopoulos, K.P.; Orr, K.; O'rourke, R.; Renwick, R.; Bruno, N.; Wright, V.; Bobbie, K.; Noronha, J. Quality of Participation Experiences in Special Olympics Sports Programs. *Adapt. Phys. Activ Q.* **2022**, *39*, 17–36. https://doi.org/10.1123/APAQ.2021-0033.

51. Liljenquist, K.; Kramer, J.; Rossetti, Z.; Coster, W. Content development, accessibility and feasibility of a self-report tool for use in programmes serving youth with cognitive disabilities: The Participatory Experience Survey. *Aust. Occup. Ther. J.* **2019**, *66*, 490–499. https://doi.org/10.1111/1440-1630.12571.
52. Liljenquist, K.; Coster, W.; Kramer, J.; Rossetti, Z. Feasibility of the Participatory Experience Survey and the Setting Affordances Survey for use in evaluation of programmes serving youth with intellectual and developmental disabilities: Feasibility of PES and SAS use in programme evaluation. *Child Care Health Dev.* **2017**, *43*, 511–517. https://doi.org/10.1111/cch.12402.
53. King, G.; Batorowicz, B.; Rigby, P.; McMain-Klein, M.; Thompson, L.; Pinto, M. Development of a Measure to Assess Youth Self-reported Experiences of Activity Settings (SEAS). *Int. J. Disabil. Dev. Educ.* **2014**, *61*, 44–66. https://doi.org/10.1080/1034912X.2014.878542.
54. Batorowicz, B.; King, G.; Vane, F.; Pinto, M.; Raghavendra, P. Exploring validation of a graphic symbol questionnaire to measure participation experiences of youth in activity settings. *Augment. Altern. Commun.* **2017**, *33*, 97–109. https://doi.org/10.1080/07434618.2017.1307874.
55. Kulis, A.; Batorowicz, B.; Chrabota, U. Validation of the Polish version of the Self-reported Experiences of Activity Settings (SEAS) questionnaire. *Rehabil. Med.* **2020**, *23*, 4–9. https://doi.org/10.5604/01.3001.0014.1510.
56. MacDonald, M.; Leichtman, J.; Esposito, P.; Cook, N.; Ulrich, D.A. The participation patterns of youth with down syndrome. *Front. Public. Health* **2016**, *4*, 253. https://doi.org/10.3389/FPUBH.2016.00253.
